# Validation of a mathematical–geometrical model to calculate the length of an individual anterior arch

**DOI:** 10.1007/s00056-023-00482-2

**Published:** 2023-07-03

**Authors:** Eva Paddenberg, Raphael Heiß, Tobias Grünbaum, Peter Proff, Christian Kirschneck

**Affiliations:** 1 Department of Orthodontics, University Hospital Regensburg, Franz-Josef-Strauß-Allee 11, 93053 Regensburg, Germany; 2 Institute for Experimental and Applied Physics, University of Regensburg, Universitätsstraße 31, 93053 Regensburg, Germany

**Keywords:** Individualised diagnostics, Orthodontic diagnostics, Anterior arch length, Cast analysis, Polynomial models, Individualisierte Diagnostik, Kieferorthopädische Diagnostik, Anteriore Zahnbogenlänge, Modellanalyse, Polynomiale Modelle

## Abstract

**Purpose:**

For resolving anterior dental crowding or spacing, it is of key interest in personalised orthodontic diagnostics and treatment planning to predict the extent of space gained or lost in the anterior dental arch by changing incisor inclination or position. To facilitate the determination of anterior arch length (AL) and to predict its alterations following tooth movements, a mathematical–geometrical model, based on a third-degree parabola, was established. The aim of this study was to validate this model and assess its diagnostic precision.

**Methods:**

This retrospective diagnostic study evaluated 50 randomly chosen dental casts taken before (T0) and after (T1) orthodontic treatment with fixed appliances. Plaster models were digitally photographed, allowing two-dimensional digital measurements of arch width, depth and length. A computer programme based on the mathematical–geometrical model to be validated was created to calculate AL for any given arch width and depth. Mean differences and correlation coefficients as well as Bland–Altman plots were used to compare the measured and the calculated (predicted) AL, evaluating the precision of the model.

**Results:**

Inter- and intrarater reliability tests showed reliable measurements of arch width, depth and length. Measured and calculated (predicted) AL revealed high concordance according to concordance correlation coefficient (CCC), intraclass correlation coefficient (ICC), and Bland–Altman analyses and negligible differences between the mean values.

**Conclusions:**

The mathematical–geometrical model calculated anterior AL without significant difference to the measured AL, indicating its validity. The model can thus be used clinically for predicting alterations of AL following therapeutic changes of incisor inclination/position.

**Supplementary Information:**

The online version of this article (10.1007/s00056-023-00482-2) contains supplementary information, which is available to authorized users.

## Abstract (de)

**Ziel:**

Zur Korrektur eines anterioren Zahnengstandes bzw. Zahnlückenstandes ist in der personalisierten kieferorthopädischen Diagnostik und Therapieplanung die Vorhersage des Platzgewinns bzw. Platzverlustes im anterioren Zahnbogen infolge einer Veränderung der Inklination und Position der Inzisiven von großem Interesse. Entwickelt wurde ein mathematisch-geometrisches, auf einer kubischen Parabelfunktion basierendes Modell, um die anteriore Zahnbogenlänge (AL) einfacher bestimmen und Dimensionsänderungen nach Zahnbewegungen vorhersagen zu können. Das Ziel dieser Studie war es, das Modell zu validieren und die diagnostische Präzision zu überprüfen.

**Methode:**

Diese retrospektive Diagnosestudie analysierte 50 randomisiert gewählte Studienmodelle, die vor (T0) und nach (T1) einer kieferorthopädischen Behandlung mit festsitzender Apparatur erstellt wurden. Zur zweidimensionalen digitalen Messung von Zahnbogenbreite, -tiefe und -länge wurden die Studienmodelle digital fotografiert. Um die AL für jede gegebene Bogenweite und -tiefe zu bestimmen, wurde ein auf dem zu validierenden mathematisch-geometrischen Modell basierendes Computerprogramm entwickelt. Durchschnittliche Differenzen, Korrelationskoeffizienten und Bland-Altman-Diagramme wurden genutzt, um die gemessene und berechnete (vorhergesagte) AL zu vergleichen und so die Präzision des Modells zu prüfen.

**Ergebnisse:**

Interrater- und Intrarater-Reliabilitätstest ergaben zuverlässige Messungen der Zahnbogenbreite, -tiefe und -länge. Die gemessene und die berechnete (vorhergesagte) AL zeigten entsprechend den CCC („concordance correlation coefficient“), ICC („intraclass correlation coefficient“) und Bland-Altman-Analysen eine hohe Konkordanz und vernachlässigbare Unterschiede zwischen den Mittelwerten.

**Schlussfolgerungen:**

Da das mathematisch-geometrische Modell die AL ohne signifikante Unterschiede zur gemessenen AL berechnete, wurde die Validität des Modells belegt. Somit kann das Modell klinisch zur Vorhersage von Veränderungen der AL infolge einer therapeutischen Veränderung der Inklination/Position der Inzisiven verwendet werden.

## Introduction

Dental arch form is often altered during orthodontic treatment due to several reasons including space conditions, mostly to resolve anterior crowding by incisor proclination. However, to achieve functional loading during static and dynamic occlusion as well as stable treatment results in the long-term, the morphological conditions of the individual patient, including the alveolar base and the corresponding ideal dental arch form, must be considered. For this purpose, dental casts and lateral cephalograms are analysed, allowing the diagnosis of space conditions within the dental arch as well as the determination of incisor inclination and position, respectively. Considering both diagnostic results, the extent and type of orthodontic movement of the anterior teeth can be decided based upon personalised orthodontic diagnostics.

Altering the dimension of the dental arch form may be indicated due to several reasons including crowding or spacing, malposition of the anterior and/or posterior teeth and malocclusion in either dimension. Especially the anterior arch is influenced by changing the position and inclination of the front teeth and by arch expansion and compression. Since dental crowding is present in 60% of the orthodontic patients in Germany [14], the orthodontist is often confronted with the question about performing changes in arch dimension when resolving dental crowding. In these cases, proclination or anterior translation of incisors is one possible therapeutic option apart from molar distalisation, changing arch width, proximal enamel reduction or tooth extraction. Knowing the variations in arch dimension and thereby space conditions may thus be useful in personalised differential treatment planning in orthodontics.

For this purpose, a mathematical–geometrical model, which individually calculates the anterior arch length (AL) for a given width and sagittal depth of the anterior arch, consisting of the canines and the incisal point, was established at the University of Regensburg by C. Kirschneck and T. Grünbaum. Since arch depth is influenced by the position of the incisal point, which can be altered therapeutically in the vertical and sagittal direction, the cephalometrically determined inclination of the incisors was also considered. Hence, this model is also expected to allow a pretherapeutic prediction of the alterations in anterior arch form and thus of space conditions, when the inclination of the incisors, arch width or arch depth are changed therapeutically. Inclination of the incisors can be determined by several angular measurements in lateral cephalograms, including *1/ML*, *1/NL*, *1/NSL*, *1/NA*, *1/NB*, which can be converted among each other, applying mathematical formulae (Supplementary Figure 1).

**Fig. 1:**
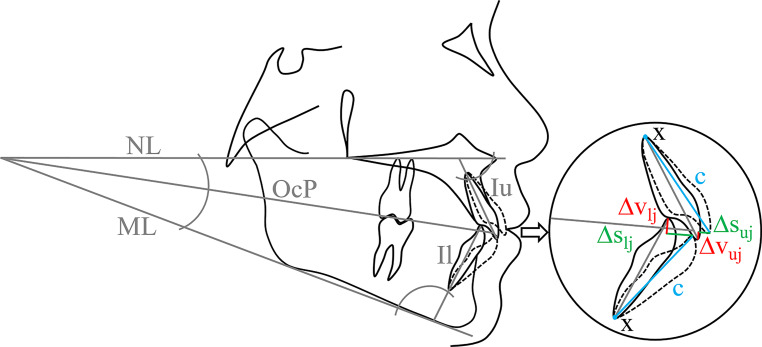
Sagittal and vertical changes of the upper and lower incisal point induced by altering incisor inclination. *Iu* tooth axis of the upper central incisor, *Il* tooth axis of the lower central incisor, *x* centre of rotation,* c* linear distance between centre of rotation and incisal edge, *ML* mandibular line, *NL* nasal line, *OcP* occlusal plane, *uj* upper jaw, *lj* lower jaw, *∆s* sagittal changes, *∆v* vertical changes Sagittale und vertikale Veränderungen der oberen und der unteren Inzisalkante infolge einer unterschiedlichen Frontzahninklination. *Iu* Zahnachse des oberen mittleren Frontzahnes, *Il* Zahnachse des unteren mittleren Frontzahnes, *x* Rotationszentrum, *c* lineare Distanz zwischen Rotationszentrum und Inzisalkante, *ML* Mandibularlinie, *NL* Nasallinie, *OcP* Okklusionsplanum, *uj* Oberkiefer, *lj* Unterkiefer, *∆s* sagittale Veränderungen, *∆v* vertikale Veränderungen

Therapeutic movements of the incisal point were subdivided into vertical (*∆v*) and sagittal (*∆s*) changes in the midsagittal plane (lateral cephalogram), and are influenced by the linear distance between the incisal edge of the central incisors and their centre of rotation (*c*; Fig. 1), which defines different types of tooth movements which can be reached mechanically from different moment-to-force ratios [29]. The situation before orthodontic treatment was denoted as T0, the one after treatment as T1.

Regarding *∆s*, positive values were defined as an anteriorly directed movement, i.e. protrusion, whereas negative values indicated a retrusion. Total sagittal changes were composed of translational (*∆s*(translation)), i.e. a bodily movement, of the incisor in sagittal direction, and tipping movements (*∆s*(tipping)), resulting in the following formulae for the upper and lower jaw, respectively.

$$\text{Upper jaw:}\ \Updelta s\left(uj{,}\textit{total}\right)=\Updelta s\left(uj{,}\textit{tipping}\right)+\Updelta s(uj{,}\textit{translation})$$

$$\text{Lower jaw:}\ \Updelta s\left(lj{,}\textit{total}\right)=\Updelta s\left(lj{,}\textit{tipping}\right)+\Updelta s(lj{,}\textit{translation})$$

Whereas *∆s*(translation) corresponds exactly to the actual amount of sagittal translational movement of the incisors, *∆s*(tipping) can be calculated using trigonometry from angular measurements, which can be derived by cephalometric analysis (Fig. 1):

$$\it \it \it \it \it \it \text{Upper jaw:}\ \Updelta s\left(uj{,}\textit{tipping}\right)=c\left(uj\right)\times (\cos (\sphericalangle 1/NL_{T0}-\sphericalangle \textit{OcP}/NL_{T0}-\left(-\Updelta \sphericalangle 1/NL_{T0/T1}\right))-\cos (\sphericalangle 1/NL_{T0}-\sphericalangle \textit{OcP}/NL_{T0}))$$

$$\it \it \text{Lower jaw:}\ \Updelta s\left(lj{,}\textit{tipping}\right)=c\left(lj\right)\times (\cos (180{^{\circ}}-\sphericalangle \textit{OcP}/ML_{T0}-\sphericalangle 1/ML_{T0}-\Updelta \sphericalangle 1/ML_{T0/T1})-\cos (180{^{\circ}}-\sphericalangle \textit{OcP}/ML_{T0}-\sphericalangle 1/ML_{T0}))$$

Alterations of the incisal point in the vertical direction were measured perpendicularly to the occlusal plane (*OcP*), passing through half overbite and the occlusal contact of the most distal tooth. Positive values of *∆v* were defined by movements of the incisal point away from *OcP*, i.e. intrusion, whereas negative values expressed incisor extrusion. Applying trigonometry, the following two equations were established to compute *∆v* for the upper and lower jaw, respectively, based on cephalometric analysis (Fig. 1):

$$\it \it \it \text{Upper jaw:}\ \Updelta v\left(uj\right)=-c\left(uj\right)\times (\sin (\sphericalangle 1/NL_{T0}-\sphericalangle \textit{OcP}/NL_{T0}-\left(-\Updelta \sphericalangle 1/NL_{T0/T1}\right))-\sin (\sphericalangle 1/NL_{T0}-\sphericalangle \textit{OcP}/NL_{T0})$$

$$\it \it \it \text{Lower jaw:}\ \Updelta v\left(lj\right)=-c\left(lj\right)\times (\sin (\sphericalangle 1/ML_{T0}+\sphericalangle \textit{OcP}/ML_{T0}+\Updelta \sphericalangle 1/ML_{T0/T1})-\sin (\sphericalangle 1/ML_{T0}+\sphericalangle \textit{OcP}/ML_{T0}))$$

Hence, the total amount of bite opening or deepening was calculated by the sum of vertical changes in the maxilla and in the mandible:

$$\Updelta v\left(\textit{total}\right)=\Updelta v\left(lj\right)+\Updelta v(uj)$$

**Fig. 2:**
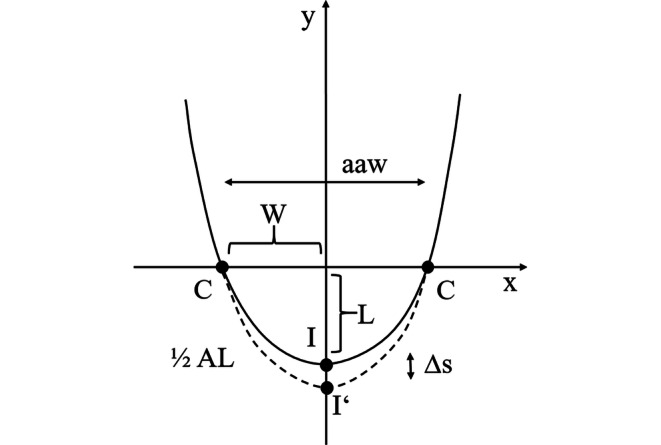
Reference points and measurements of the anterior arch form placed in a two-dimensional coordinate system before (*solid line*) and after (*dashed line*) treatment. *I* incisal point before orthodontic treatment; *I’* incisal point after orthodontic treatment (anterior or posterior translation and/or tipping of incisors); y‑axis corresponds to raphe median plane (RMP), x‑axis to the posterior border of the anterior arch; *aaw *anterior arch width; *W* ½ aaw; *C* distal contact point of the canine; *L* sagittal depth of the anterior arch; *AL* length of the anterior arch; *∆s* sagittal changes of the anterior arch Referenzpunkte und Messungen der anterioren Zahnbogenform unter Projektion in ein zweidimensionales Koordinatensystem vor und nach der Behandlung. *I* Inzisalkante vor kieferorthopädischer Behandlung, *I’* Inzisalkante nach kieferorthopädischer Behandlung (anteriore oder posteriore Translation und/oder Kippung der Frontzähne), die y‑Achse entspricht der Raphe-Median-Ebene (RMP), die x‑Achse entspricht der posterioren Begrenzung des anterioren Zahnbogens, *aaw* anteriore Zahnbogenbreite, *W* ½ aaw, *C* distaler Kontaktpunkt des Eckzahnes, *L* sagittale Tiefe des anterioren Zahnbogens, *AL* Länge des anterioren Zahnbogens, *∆s* sagittale Veränderungen des anterioren Zahnbogens

These assumptions about the current cephalometrically derived position and the aimed therapeutic movement of the incisal point are included in the actual mathematical–geometrical model about anterior arch form. For simplification purposes, movements of the incisal point were projected onto the horizontal plane *OcP*. The model is based on a third-degree polynomial, which is defined as the sum of multiple terms containing a variable with a maximum power of three, because Schumacher et al. [26, 27] concluded from anatomical–morphologic studies that a third-degree parabola is most suitable to describe anterior arch form (Fig. 2). Since the third-degree parabola is point-symmetrical, its right positive side was reflected at the y‑axis to achieve a U-shape of the anterior arch. The line connecting the distal contact points of the canines was placed at the origin of the two-dimensional (2D) Cartesian coordinate system, i.e. on the x‑axis, and the incisal point, i.e. the vertex of the parabola, was moved in negative direction of the y‑axis by the amount corresponding to the anterior arch length. Half transverse width (*W*) of the anterior arch was defined as the linear distance between the distal contact point of the canine and the raphe median plane (RMP), perpendicular to RMP. Sagittal depth (*L*) of the anterior arch was measured as the linear distance between the buccal surface of the central incisors and the line used to determine *W*, perpendicular to *W* (Fig. 2).

Taking the above information into account, the following formula to define the parabolic shape of the dental arch was used as a basis of the mathematical–geometrical model to describe *AL*:

$$\mathrm{y}=a\mathrm{x}^{3}-L$$

As the formula clarifies, it is influenced by the coefficient *a*, which describes the openness factor of the parabola and expresses the width of the anterior arch form. The smaller *a*, the wider the parabola and thus also the dental arch. To determine the value of *a*, the formula can be rearranged for y = 0, i.e. for the arch width at the distal contact point of the canine (*x* = *C* = (*aaw*/2)): *a* = *L* / (*aaw*/2)^3^ = *L* / *W*^3^, where *C* is the distal contact point of the canine. Then the parabola of the dental arch can be expressed as the following function:

$$f\left(x\right)=\frac{L}{\left(\frac{aaw}{2}\right)^{3}}x^{3}-L$$

Next, arch length can be calculated using line integration, which includes the first derivative function of the above expression, *f’(x)*:

$$\text{Arch length}\left(\mathrm{t}\right)=2\times {\int }_{0}^{\mathrm{t}}\sqrt{1+\left(f\mathrm{'}\left(\mathrm{x}\right)\right)^{2}} d x$$

Using the integration limits *0* and *t*, only half of the arch length is calculated and hence the integral must be multiplied by the factor “2” to obtain the full arch length, as stated in the formula. The upper integration limit *t* represents the x‑value, where the function *f(x)* cuts the x‑axis and therefore corresponds to the distal contact point of the canine, representing the posterior boundary of the arch.

Finally, the first derivative of the expression *f(x), f’(x)*, and the upper integration limit *aaw*/2 must be inserted into the formula, resulting in the following equation to calculate the initial pretherapeutic anterior arch length *AL* of a given patient based on the parameters anterior arch width (*aaw*) and sagittal depth of the anterior arch (*L*):

$$\text{Arch length}\,(\mathrm{AL})_{\mathrm{T}0} = 2\times {\int }_{0}^{\frac{aaw}{2}} \sqrt{1+9 \frac{L^{2}} {\left(\frac{aaw}{2}\right)^{6}} x^4} d x$$

If changes of incisor inclination or their translation in the sagittal direction are planned for therapeutic reasons, that is, if the incisal point is moved in sagittal direction (*∆s*), the resulting new posttherapeutic anterior arch length *AL* can be predicted through the following mathematical equation:

$$\text{Arch length}\,\left(\mathrm{AL}\right)_{\mathrm{T}1} =2\times {\int }_{0}^{\frac{\mathrm{aaw}_{T1}} {2} } \sqrt{1+9 \frac{\left(L_{T0}+\Updelta s\left(\textit{total}\right)\right){^{2}}} {\left(\frac{\mathrm{aaw}_{T0}+\Updelta aaw}{2}\right){^{6}}} x^4} dx$$

The therapeutic change in dental arch length can thus be calculated as:

$$\Updelta AL_{T0/T1}=AL_{T1}-AL_{T0}$$

If not only the incisal point, but also the anterior arch width (*aaw*) is altered during treatment (*∆aaw*), the upper limit of the integral must be changed and the openness factor *a* must be adapted (*a’*) in the following way:

$$\mathrm{a}\mathrm{'}=\frac{L_{T1}}{\left(\frac{\mathrm{aaw}_{T1}}{2}\right)^{3}}=\frac{L_{T0}+\Updelta s\left(\textit{total}\right)}{\left(\frac{\mathrm{aaw}_{T0}+\Updelta aaw}{2}\right)^{3}}$$

The presented mathematical–geometrical model to predict changes in anterior arch length pretherapeutically only has value if it actually conforms to and accurately reflects the clinical situation. Thus, it needs to be validated based on clinical patient cases which were treated by incisor protrusion, retrusion or translation to assess whether the changes in dental arch length *∆AL* predicted by the model were actually achieved posttherapeutically.

The aim of this retrospective cross-sectional study was therefore to present and to validate the mathematical–geometrical model conducted for the calculation of individual anterior arch length by evaluating pre- and posttreatment dental casts and lateral cephalograms of orthodontic patients and to assess its diagnostic precision. Further investigations are planned to implement this knowledge in personalised treatment planning, facilitating the prognosis of changes in anterior arch dimension by altering the inclination or position of incisors, arch width or arch depth.

## Materials and methods

This retrospective diagnostic study was based on dental casts and lateral cephalograms of 50 randomly selected orthodontic patients who were treated with fixed appliances in the upper and lower jaw between 2015 and 2017 at the Department of Orthodontics at the University Hospital Regensburg, Germany. All casts were anonymised directly at the source by a study number. T0 and T1 were defined as the times pre- and posttreatment, respectively. Patients were considered for inclusion if they presented plaster models of good quality with precise reproduction of the arches free of distortion before and after orthodontic treatment with fixed appliances. Exclusion criteria were patients with cleft lip and palate, craniofacial syndromes, tooth agenesis, anomaly of the number or form/structure of teeth and front teeth, which were not fully erupted for clear measurements to allow precise measurements without any distortion.

To analyse anterior arch dimensions, the upper and lower arch of each patient were evaluated before (T0) and after (T1) orthodontic treatment. For this purpose, diagnostic casts were trimmed in a manner that their base area was parallel to the orthodontic occlusal plane, passing through the buccal cusp of the first premolars and mesiobuccal cusp of the first molars in the maxilla. RMP was identified in the upper jaw by a line passing through the crossing point between the second pair of palatal rugae and the anatomic median palatine raphe and the midpoint of the fovea palatina, and then transferred to the lower jaw. Next, each cast was put down on the base area and a glass plate with a reticule was placed on the arch, so that the vertical line of the reticule was congruent to RMP. Then, a digital occlusal photograph (Canon EOS 500D, Canon Inc., Tokyo, Japan) was taken, keeping the reticule on the glass plate congruent to the anatomical reference lines on the cast to avoid parallax errors and to project the three-dimensional (3D) arch into a 2D area parallel to the orthodontic occlusal plane, that can be analysed digitally. By focusing on the reticule, all photographs were taken with the same setting, focusing on the occlusal plane. To allow calibration of the photographs without distortion to perform metrical measurements, a ruler was placed next to the plaster model during photographing.

**Fig. 3:**
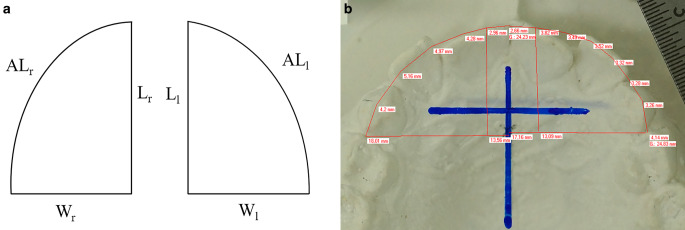
**a**–**b** Anterior arch length. **a** Variables measured on the digital photographs of the dental casts: *AL* anterior arch length, *W* half arch width, *L* sagittal depth, *l* left, *r* right. **b** Example of a digital photograph showing the measurements performed (*AL*, *W*, *L* for both sides) by approximating the ideal arch by 12 linear subsections (*red*) Anteriore Zahnbogenlänge AL. **a** Variablen, die auf den digitalen Fotos der Situationsmodelle gemessen werden: *AL* anteriore Zahnbogenlänge, *W* halbe Zahnbogenbreite, *L* sagittale Tiefe, *l* links, *r* rechts. **b** Beispiel eines digitalen Fotos, das die durchgeführten Messungen am idealen Zahnbogen, welcher durch 12 lineare Abschnitte angenähert wird (*rot*), zeigt (*AL*, *W*, *L* für beide Seiten)

Next, photographs were imported as lossless TIF files into the online-accessible computer programme “Aufmaßprogramm” (Jens Rüdig; https://ruedig.de/tmp/messprogramm.htm) and calibrated. Then, linear distances were measured digitally according to the following procedure: in each photograph the half arch width (*W* = *aaw*/2) was measured separately for the left and right side. Afterwards, sagittal depth (*L*) of the anterior arch was determined separately for the left and right side. Finally, arch length (*AL*) was assessed separately for the left and right side by approximating the anterior arch through 12 linear subsections resulting from 13 randomly set reference points. In total, three parameters *L, W* and *AL*/2 were determined separately for the left and right side, for the upper and lower jaw at T0 and T1, resulting in 24 measurements per patient (Fig. 3). These measurements were repeated three times and the corresponding mean value was used for further analyses to reduce measurement error.

The anterior arch form was approximated by straight lines, and hence the potential error induced by this method must be evaluated. For this purpose, a black coloured arch form was approximated by different numbers of lines in the “Aufmaßprogramm” by using 5, 7, 9, 11, 13 and 15 reference points to be connected by red coloured straight lines and then added by the programme. Visual inspection revealed that 15 reference points and 14 subsections (red) covered the arch almost completely. Thus, percentage deviation of the sum of the subsections from the ideal approximation through 15 reference points and 14 linear subsections was calculated.

Based on the mathematical–geometrical model of Kirschneck and Grünbaum, a computer programme called “Prognostic calculator for orthodontic corrections of the anterior dental segment” was established using the programming language Java (Oracle Corporation, Redwood City, CA, USA) by C. Kirschneck and M. Winter. This way, arch length, depth and width as well as cephalometric variables concerning the incisors’ inclination and position could be easily calculated according to the formulae established. *AL* was calculated for any given *aaw* and *L* at T0 and T1, and then compared to the respective measured *AL* at the plaster cast to validate the mathematical–geometrical model. Means of the measurements arch width (*aaw* = 2 × *W*) and arch depth (*L*), which were determined three times on the digital photographs, were entered in the programme and *AL* was computed. Like the measured parameters, *AL* calculation was performed separately for the left and right side of each arch, entering the corresponding measured width and depth. Furthermore, total length of the anterior arch was calculated by entering the mean of the left and right sagittal depth (*L*_total_ = (*L*_left_ + *L*_right_) / 2) and the sum of the left and right arch width (*W*_total_ = *aaw* = *W*_left_ + *W*_right_). The reference *AL*_measured_ was the sum of the left and right AL (*AL*_measured total_ = *AL*_measured left_ + *AL*_measured right_). In total, for each lower and upper arch at T0 and T1 three measured (left, right, total) and three calculated (left, right, total) *AL* values were calculated, resulting in 24 *AL *values per patient.

Statistical analysis was performed using the software SPSS (IBM SPSS Statistics 22, Armonk, NY, USA) and the online-accessible NIWA tool for the concordance correlation coefficient (CCC; https://www.niwa.co.nz/node/104318/concordance). Intra- and interrater reliability were tested for the measured parameters *W*_right_, *W*_left_, *L*_right_, *L*_left_, *AL*_right_ and *AL*_left_, using intraclass correlation coefficient (ICC) and Lin’s CCC [19], respectively. Interrater reliability testing was based on 20 randomly selected patients and performed independently by two different raters (RH, CK), whereas intrarater reliability testing was performed by evaluating all 50 patients three times with a time interval of at least 2 weeks by the same rater (RH). Interpretation of the correlation coefficients followed the cut-off limits of Fleiss et al. [12] and Koch and Spörl [18] for the ICC and CCC, respectively. Descriptive statistics were conducted for all measured variables, i.e. *L, W, AL*_measured_, and for the calculated parameter *AL*_calculated_, including mean, standard deviation, minimum, maximum and 95% confidence interval (CI). For analytical statistics, the differences between the measured and calculated AL values were computed to check precision of the calculated *AL*. To clarify clinical relevance of the difference, it was given in mm, since correlation coefficients allow abstract estimations only. To avoid distortion through positive and negative differences, only absolute values were computed. Hence, the extent of the difference was better indicated without analysing the direction of the deviation. Furthermore, the correlation between the measured and calculated *AL *values was assessed using CCC and Bland–Altman plots. Percentage deviation between an ideal arch and its approximation by 12 linear subsections was evaluated. Finally, the error induced by false identification of the *OcP* was estimated by calculating the changes in *AL *induced by a certain incisor protrusion for different inclinations of *OcP*, keeping *ML-NL* fixed.

## Results

Intrarater reliability proved excellent concordance for all measurements (ICC > 0.9). Although interrater reliability was better in the upper arch, it revealed at least an acceptable concordance between the two sets of measurements also in the lower arch (CCC > 0.5).

Measuring and calculating the arch lengths was conducted under blinding of the corresponding results.

Table 1 shows the descriptive statistics of the measured and calculated (predicted) variables of the upper and lower arch at T0 and T1. The results indicate that the corresponding measured and calculated (predicted) arch lengths were comparable, regardless of the jaw and timepoint.

**Table 1:** Descriptive statistics for the measured parameters *L*, *W* and *AL* and for the calculated (predicted) *AL* of the upper and lower arch at T0 and T1. Measured variables represent the mean of three repeated measurements per patient. All measurements are given in mm. *N* = 50 Deskriptive Statistik der gemessenen Parameter *L*, *W* und *AL* sowie der (prognostizierten) *AL* des oberen und des unteren Zahnbogens zu T0 und T1. Die gemessenen Variablen repräsentieren den Mittelwert von 3 wiederholten Messungen pro Patient. Alle Messungen sind in mm angegeben. *N* = 50

	Arch		Upper arch				Lower arch			
Time	Variable		Mean ± SD	Min	Max	95% CI	Mean ± SD	Min	Max	95% CI
T0	*L*_*measured*_	*r*	13.9 ± 2.2	7.7	18.3	13.3; 14.5	9.0 ± 2.0	4.9	14.1	8.5; 9.6
		*l*	14.0 ± 2.0	9.8	17.9	13.5; 14.6	9.1 ± 1.5	5.9	12.9	8.7; 9.5
		*(r* *+* *l) /2*	14.0 ± 2.0	9.0	18.1	13.4; 14.5	9.1 ± 1.6	5.9	13.4	8.6; 9.5
	*W*_*measured*_	*r*	18.2 ± 1.5	13.3	20.5	17.8; 18.6	14.9 ± 1.3	12.0	17.8	14.5; 15.3
		*l*	18.1 ± 1.4	13.3	20.6	17.7; 18.4	14.8 ± 1.4	12.2	17.3	14.5; 15.2
		*r* *+* *l*	36.2 ± 2.6	26.6	41.1	35.5; 37.0	29.7 ± 2.2	25.3	34.2	29.1; 30.4
	*AL*_*measured*_	*r*	24.8 ± 1.9	21.9	28.7	24.3; 25.4	19.2 ± 2.1	15.7	25.1	18.6; 19.8
		*l*	25.3 ± 2.1	20.3	29.5	24.7; 25.9	18.8 ± 1.6	16.2	23.7	18.3; 19.2
		*r* *+* *l*	50.1 ± 3.8	43.7	57.3	49.1; 51.2	38.0 ± 3.2	32.5	47.2	37.1; 38.9
	*AL*_*calculated*_	*r*	24.8 ± 2.0	20.3	29.0	24.2; 25.3	18.7 ± 2.0	15.4	24.5	18.2; 19.3
		*l*	24.9 ± 2.1	19.8	29.2	24.3; 25.5	18.8 ± 1.6	15.1	23.3	18.2; 19.1
		*r* *+* *l*	49.7 ± 4.0	41.5	57.2	48.6; 50.8	37.4 ± 3.2	32.1	46.8	36.5; 38.3
T1	*L*_*measured*_	*r*	13.5 ± 1.5	10.5	16.6	13.3; 14.2	9.1 ± 1.4	6.6	12.7	8.8; 9.5
		*l*	13.6 ± 1.4	10.9	17.0	13.2; 14.0	9.0 ± 1.4	6.5	12.0	8.6; 9.3
		*(r* *+* *l) /2*	13.7 ± 1.4	10.8	16.6	13.3; 14.1	9.1 ± 1.2	6.8	12.0	8.7; 9.4
	*W*_*measured*_	*r*	18.8 ± 1.0	16.0	21.3	18.5; 19.1	15.2 ± 0.9	13.0	17.4	14.9; 15.4
		*l*	18.8 ± 1.1	16.1	21.6	18.5; 19.1	15.0 ± 0.9	13.2	16.8	14.8; 15.3
		*r* *+* *l*	37.6 ± 1.9	32.1	42.2	37.1; 38.2	30.2 ± 1.5	26.3	33.4	29.8; 30.6
	*AL*_*measured*_	*r*	25.1 ± 1.6	21.4	28.9	24.7; 25.6	19.4 ± 1.4	16.3	22.4	19.0; 19.8
		*l*	25.7 ± 1.6	22.0	29.4	25.2; 26.1	18.7 ± 1.3	16.5	21.5	18.3; 19.1
		*r* *+* *l*	50.8 ± 3.2	43.4	58.2	49.9; 51.7	38.1 ± 2.4	32.8	43.0	37.4; 38.8
	*AL*_*calculated*_	*r*	25.3 ± 1.7	22.1	29.4	24.8; 25.7	19.0 ± 1.5	15.9	22.3	18.6; 19.4
		*l*	25.1 ± 1.6	21.9	28.3	24.7; 25.5	18.7 ± 1.3	16.7	21.4	18.4; 19.1
		*r* *+* *l*	50.3 ± 3.1	44.0	57.7	49.5; 51.2	37.8 ± 2.5	33.2	43.1	37.1; 38.4

*T0* pretreatment, *T1* posttreatment, *L* sagittal depth of the anterior arch, *W* width of the anterior arch, *AL* anterior arch length, *r* right, *l* left, *SD* standard deviation, *Min* minimum, *Max* maximum, *CI* confidence interval

The absolute value of the difference between the measured and calculated *AL* is presented in Table 2. On average, the largest difference was 0.7 mm and the standard deviations were smaller than 0.6 mm, showing that both methods are equally appropriate to determine anterior arch length.

**Table 2:** Descriptive statistics of the absolute value of the difference between measured and calculated (predicted) arch length of the upper and lower arch at T0 and T1. All measurements in mm. *N* = 50 Deskriptive Statistik des Betrages aus der Differenz zwischen gemessener und berechneter (prognostizierter) Zahnbogenlänge im oberen und im unteren Zahnbogen zu T0 und T1. Alle Messungen sind in mm angegeben. *N* = 50

	Arch	Upper arch			Lower arch		
Time	Statistic	| AL measured − AL calculated |			| AL measured − AL calculated |		
		r	l	t	r	l	t
T0	Mean	0.36	0.60	0.70	0.56	0.33	0.66
	SD	0.29	0.41	0.57	0.34	0.27	0.46
	Max	1.54	1.79	2.64	1.71	1.29	1.93
	Min	0.01	0.00	0.02	0.06	0.00	0.06
	95% CI	0.28–0.44	0.48–0.71	0.54–0.86	0.46–0.65	0.26–0.41	0.53–0.79
T1	Mean	0.32	0.56	0.51	0.43	0.18	0.39
	SD	0.29	0.30	0.31	0.26	0.14	0.28
	Max	1.73	1.09	1.1	1.02	0.53	1.34
	Min	0.00	0.1	0.01	0.01	0.01	0.00
	95% CI	0.24–0.40	0.48–0.65	0.43–0.60	0.35–0.50	0.14–0.22	0.31–0.47

*T0* pretreatment, *T1* posttreatment, *SD* standard deviation, *Max* maximum, *Min* minimum, *CI* confidence interval, *AL* arch length, | *AL* measured − *AL* calculated | = norm of the difference, *r* right, *l* left; *t* total

The CCC ranged from 0.924 to 0.985 with a mean CCC of 0.962, thereby showing the high validity of the mathematical–geometrical model, as concordance was substantial.

Bland–Altman plots revealed as well good concordance between the measured and calculated *AL *(Supplementary Figures 2 and 3).

The percentage differences between the arch length of the ideal arch (14 subsections) and the approximated arch revealed that the use of 10 and 12 subsections caused only a negligible difference of 0.9% and 0.5%, respectively.

The error induced by false identification of the *OcP* up to 6° was below 0.1 mm and hence was not relevant for the clinical validity of the mathematical model (Supplementary Table 1).

## Discussion

This retrospective study was conducted to present and to validate a mathematical–geometrical model of C. Kirschneck and C. Grünbaum to compute anterior arch length of an individual patient, if the width and sagittal depth of the anterior arch are known and the position of the incisal point is altered therapeutically in the sagittal direction, thus changing the anterior dental arch curvature. For this purpose, a computer programme was developed to calculate the arch length based on the mathematical model and 2D digitised photographs of dental casts were evaluated to acquire measured arch length as a reference parameter.

According to the ICC values, intrarater reliability of measuring the depth, width and length of the anterior arch showed reproducible measurements. Hence, instead of repeating each measurement three times, single measurements seem to be sufficient in daily routine, being also less time-consuming. As the results of the CCC illustrate, interrater reliability of these assessments was good. A subsequent evaluation revealed that in some cases minor concordance in the lower arch resulted from distinct malocclusions or in cases where the reference arch form was determined differently by the two raters. However, when the mathematical–geometrical model is used, only the width and depth of the anterior arch must be assessed, which may be less affected by this source of error.

As the descriptive statistics of the arch parameters demonstrate, standard deviations of the measured variables depth, width and length were relatively small, which indicates that all cases analysed presented similar dimensions. This is not surprising, because by applying the exclusion criteria, patients with major deviations to be expected, e.g. cleft lip and palate, were not considered for eligibility in our study.

Applying analytical statistics, the concordance between the measured and calculated arch length and thereby the validity of the mathematical–geometrical model was evaluated. According to the absolute value of the difference between these two lengths and to CCC comparing measured and calculated arch length, the newly developed mathematical–geometrical model proved to be valid to predict individual arch length.

The mathematical–geometrical model seems to be appropriate to describe the anterior arch length of an individual patient, regardless of the arch shape. The adaptability of the model becomes obvious, when the formula is inspected: the opening coefficient *a*, which expresses the slope of the parabola, is directly affected by the sagittal depth and the width of the anterior arch and thus, variations in either dimension of the 2D arch are considered in the equation. Furthermore, the applicability of the model was not influenced by the timepoint in terms of the pre- or posttreatment situation, since both casts revealed concordance between the measured and calculated arch length.

In the literature there are other mathematical–geometrical models that describe the dental arch form. Several publications about the (anterior) arch form are based on conic sections [1, 3, 4, 11, 15, 28, 32] which may have the characteristics of a circle, an ellipse, a parabola or a hyperbola. To describe the anterior arch form, Hawley [15] developed a geometrical method by drawing a circle with the radius equalling the mesiodistal widths of the incisors and canines. An equilateral triangle is then constructed from the circle and its edge length is used as the radius to construct a second bigger circle that allows the establishment of a second equilateral triangle, whose posterior cross points with the circle represent the end of the arch. Other approaches were based on a circle [28, 32], a semi-ellipse [4] or a parabola [1]. These attempts can be refined by a computer programme, which transforms the coordinates of the reference points on the 2D occlusal plane to characterise the individual arch form by one of the three curves as parabolic, hyperbolic or ellipsoidal [3]. Another method to describe the arch form is the catenary curve [9, 21], where the length of the chain and the distance between the suspension points are adopted as the individual arch form. This geometrical concept can be expressed by the mathematical formula of a hyperbolic cosine. Also the concept of the cubic spline curve, which can be applied in a computer programme with some fixpoints determined on dental casts was suggested to express dental arch form [2]. Other authors used a polynomial to determine an individual arch shape. Whereas Schumacher et al. [27] concluded that a third-degree parabola is most suitable, Lu [20] found a fourth-degree orthogonal polynomial appropriate to approach the arch form. Noroozi et al. [23] regarded a sixth-degree polynomial to calculate arch length with a computer programme if the depth and width at the canines and second molars are known. Also Pepe [25] ascribed highest accuracy to a sixth-degree polynomial, but claimed that neither catenary nor polynomial functions were good methods to describe the arch form. To increase the precision in arch form approximation, Valenzuela et al. [31] modified the Fourier series, which is defined as the sum of sine and cosine functions. According to their results, this technique was more concise than the fourth-degree polynomial. Braun et al. [6] evaluated several mathematical functions and found that a beta function was most suitable to describe the individual arch shape. But, when evaluating the effect of arch width expansion on arch length, keeping its depth fixed, Hnat et al. [16] stated that the beta function was appropriate to describe only the expanded arch posterior to the canines, whereas the anterior section was better approximated by the hyperbolic cosine function. Moreover, comparing polynomial and beta function, Noroozi et al. [24] concluded that a sixth degree polynomial was as appropriate as a beta function to describe the arch form and even superior for expanded arches mirroring a square form. In contrast, other authors found that the fourth degree polynomial is more appropriate to describe an individual arch than the modified beta function [17]. All the above mentioned examples illustrate that there may be more than one possible approach to describe the individual arch form, and the model presented in this study is just one. However, in contrast to some other methods [2], our technique is only used for the anterior arch form. Overall, compared to previously published ones, the advantage of our mathematical–geometrical model is that it is based on the anatomical–morphologic examinations of Schumacher et al. [26, 27] and not on a mathematical model only.

Knowing the alterations of the anterior arch dimension prior to actual orthodontic treatment is useful for treatment planning. For example, any changes in space conditions can be predicted, increasing the precision in differential personalised treatment planning, e.g. in cases where decisions about tooth extractions are necessary. According to the results of Mutinelli et al. [22], who evaluated the arch lengths following incisor advancement/protrusion in different arch forms (catenary and the cone sections circumference, ellipse, parabola, hyperbola), the alterations in arch dimensions depend on the individual arch shape. Another clinical benefit refers to the intermaxillary relationship. A computer programme or a nomogram based on the hyperbolic cosine function demonstrated that because the mesiodistal tooth masses of the incisors and canines of the upper and lower jaw (i.e. the arch perimeter) and a given arch width correlate with a certain depth, the corresponding overjet can be predicted or the ideal tooth sizes for a given arch width and depth/overjet can be proposed [7, 8]. These examples illustrate the necessity to implement the individual arch form in treatment planning. However, further factors as stability of the planned treatment and its practicability must also be considered. For example, even if a transverse expansion of the arch form may be desirable after calculating changes in arch dimension, it must be kept in mind that this treatment is at high risk of relapse [13], especially in the mandible [30]. Furthermore, when planning to change incisor inclination, the necessary moment-to-force ratios, including the centres of resistance and rotation as well as the force applied, must be considered, because different movements can occur [29], affecting the 3D position of the incisal point and thereby the arch dimension. Our presented model allows this consideration of the type of incisor movement based on the centre of resistance and on the analysis of a pretreatment lateral cephalogram.

As the results show, the mathematical model and thus the developed computer programme are a suitable method to calculate alterations of the anterior arch following certain orthodontic tooth movements. Although this digital tool was primarily designed for application during diagnostics and treatment planning, it could also aid the evaluation of progress during orthodontic treatment. Since partially automated digital measurements allow reproducible and effective analyses of dental casts [10], the software has the potential to increase diagnostic precision and efficacy.

A limitation of this study is the applied method to measure the arch length: the arch was approximated from the inside by adding 12 linear subsections and hence deviations from the actual arch were possible. However, according to the very small percentage difference between the ideal and approximated arch form, this potential error can be neglected. Another limiting factor is that the measured arch length was used as a reference value, although we cannot clearly evaluate whether the measured and thus the calculated arch really matched the actual anterior arch form. Also the calculated arch length must be evaluated with caution because it refers to an ideal mathematical model, which may show discrepancies from the natural arch. Another limitation is that we did not distinguish between Angle classes I, II, and III, although previous investigations found differences in the arch width and depth for these malocclusions [6]. Indeed, Braun et al. [6] considered the total arch and not only the anterior part, but future investigations should record the type of malocclusion and perform subgroup analyses to address this potential confounder. Similarly, craniofacial pattern may influence the individual arch form and should be evaluated as a possible confounder in future studies, since previous investigations revealed, at least for the upper arch, some association with the facial type [17]. Finally, surrounding soft tissues and muscles may act as a confounding factor, since they affect the arch width and depth and consequently also the arch form [5]. However, as the arch length is calculated from a given arch width and depth, which already ‘incorporate’ the muscular effects, the soft tissues can be neglected in the evaluation of the current situation.

Our results, that is the mathematical–geometrical model for *AL* calculation, can be generalised for the majority of orthodontic patients. However, since we excluded patients with craniofacial syndromes, cleft lip and palate, tooth agenesis, anomalies in tooth form/structure and incisors that were not fully erupted, the model may end up with some discrepancies in such cases.

## Conclusions

The presented mathematical–geometrical model appears to be suitable to calculate anterior arch length from a given arch width and depth for an individual patient as well as changes in arch length resulting from therapeutic alteration of incisor inclination and position. Thus, in future investigations, the arch length resulting from specific alterations of the arch width and incisor inclination and position should be predicted prior to the beginning of treatment. This would mean a further step in personalised differential orthodontic treatment planning, especially when evaluating individual space conditions.

## Supplementary Information

Supplementary figures and tables
