# Supplementary material for: Validation of a mathematical–geometrical model to calculate the length of an individual anterior arch
Source: J Orofac Orthop. 2023 Jul 3;86(2):108–18. doi: 10.1007/s00056-023-00482-2 (PMC11861399; doi:10.1007/s00056-023-00482-2)
Supplement: Supplementary file 1 — Supplementary figures and tables [file 56_2023_482_MOESM1_ESM.pdf]

Supplementary Figure 1 Conversion formulas for calculating incisor inclination applying geometry. T0 = before treatment, T1 = after treatment

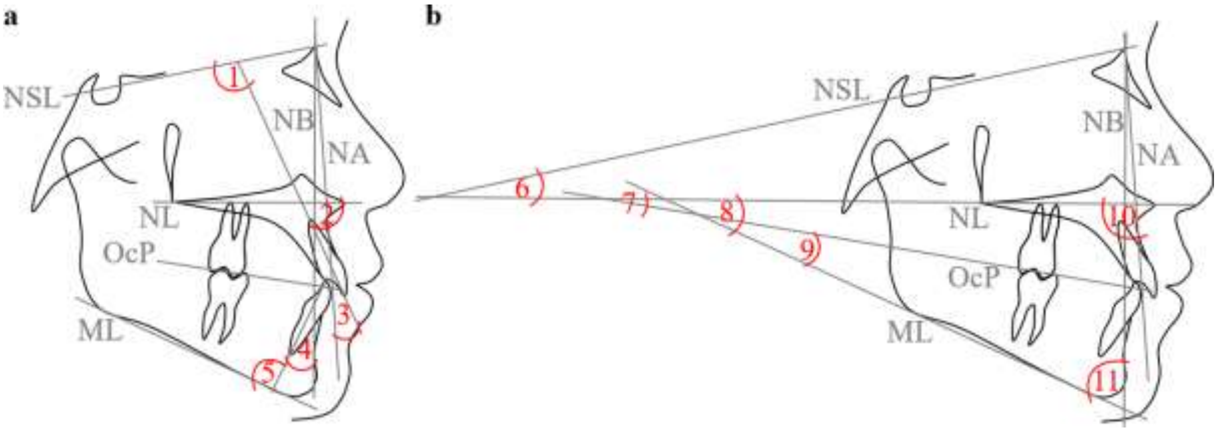

Cephalometric angular measurements of the incisors' inclination (**a**) and the reference planes (**b**). 1 = $\sphericalangle 1/NSL$, 2 = $\sphericalangle 1/NL$, 3 = $\sphericalangle 1/NA$, 4 = $\sphericalangle 1/NB$, 5 = $\sphericalangle 1/ML$, 6 = $\sphericalangle NL/NSL$, 7 = OcP/NL, 8 = ML/NL, 9 = OcP/ML, 10 = NL/NA, 11 = NB/ML

Inclination of the upper incisors

$\sphericalangle 1/NL_{T0} = 180° - \sphericalangle 1/NSL_{T0} - \sphericalangle NL/NSL_{T0}$

$\sphericalangle 1/NL_{T0} = 180° - \sphericalangle NL/NA_{T0} - \sphericalangle 1/NA_{T0}$

$\sphericalangle 1/NSL_{T0} = 180° - \sphericalangle 1/NL_{T0} - \sphericalangle NL/NSL_{T0}$

$\sphericalangle 1/NSL_{T0} = \sphericalangle NL/NA_{T0} + \sphericalangle 1/NA_{T0} - \sphericalangle NL/NSL_{T0}$

$\sphericalangle 1/NA_{T0} = 180° - \sphericalangle 1/NL_{T0} - \sphericalangle NL/NA_{T0}$

$\sphericalangle 1/NA_{T0} = -\sphericalangle NL/NA_{T0} + \sphericalangle 1/NSL_{T0} + \sphericalangle NL/NSL_{T0}$

Changes of the upper incisors' inclination

$\Delta\sphericalangle 1/NL = -\Delta\sphericalangle 1/NSL$

$\Delta\sphericalangle 1/NA_{T0} = -\Delta\sphericalangle 1/NL_{T0}$

$\sphericalangle 1/NL_{T1} = \sphericalangle 1/NL_{T0} - (-\Delta\sphericalangle 1/NL)$

$\sphericalangle 1/NSL_{T1} = 180° - \sphericalangle 1/NL_{T0} + (-\Delta\sphericalangle 1/NL) - \sphericalangle NL/NSL_{T0}$

$\sphericalangle 1/NSL_{T1} = \sphericalangle 1/NSL_{T0} + \Delta\sphericalangle 1/NSL$

$\Delta\sphericalangle 1/NSL = -\Delta\sphericalangle 1/NL$

$\Delta\sphericalangle 1/NSL = \Delta\sphericalangle 1/NA$

$\sphericalangle 1/NA_{T1} = 180° - \sphericalangle 1/NL_{T0} + (-\Delta\sphericalangle 1/NL) - \sphericalangle NL/NA_{T0}$

$\sphericalangle 1/NA_{T1} = \sphericalangle 1/NA_{T0} + \Delta\sphericalangle 1/NA$

$\Delta\sphericalangle 1/NA = -\Delta\sphericalangle 1/NL$

$\Delta\sphericalangle 1/NA = \Delta\sphericalangle 1/NSL$

Inclination of the lower incisors

$\sphericalangle 1/ML_{T0} = \sphericalangle NB/ML_{T0} + \sphericalangle 1/NB_{T0}$

$\sphericalangle 1/NB_{T0} = \sphericalangle 1/ML_{T0} - \sphericalangle NB/ML_{T0}$

Changes of the lower incisors' inclination

$\Delta\sphericalangle 1/NB = \Delta\sphericalangle 1/ML$

$\sphericalangle 1/NL_{T1} = \sphericalangle 1/NL_{T0} - (-\Delta\sphericalangle 1/NL)$

$\sphericalangle 1/ML_{T1} = \sphericalangle 1/ML_{T0} + \Delta\sphericalangle 1/ML$

$\sphericalangle 1/NB_{T1} = \sphericalangle 1/ML_{T0} + \Delta\sphericalangle 1/ML - \sphericalangle NB/ML_{T0}$

$\sphericalangle 1/NB_{T1} = \sphericalangle 1/NB_{T0} + \Delta\sphericalangle 1/NB$

$\Delta\sphericalangle 1/NB = \Delta\sphericalangle 1/ML$

Interincisal angle

$\sphericalangle 1/1_{T0} = 180° - \sphericalangle ML/NL_{T0} - \sphericalangle 1/ML_{T0} + \sphericalangle 1/NL_{T0}$

$\sphericalangle 1/1_{T0} = 180° - (\sphericalangle OcP/ML_{T0} + \sphericalangle OcP/NL_{T0}) - \sphericalangle 1/ML_{T0} + \sphericalangle 1/NL_{T0}$

Changes of the interincisal angle

$\sphericalangle 1/1_{T1} = 180° - \sphericalangle ML/NL_{T0} - \sphericalangle 1/ML_{T0} - \Delta\sphericalangle 1/ML + \sphericalangle 1/NL_{T0} - (-\Delta\sphericalangle 1/NL)$

$\Delta\sphericalangle 1/1 = - (-\Delta\sphericalangle 1/NL + \Delta\sphericalangle 1/ML)$

Supplementary Figure 2 Bland-Altman plots showing the difference between calculated and measured arch length (AL) in the upper jaw. **a**: right AL at T0, **b**: left AL at T0, **c**: total AL at T0, **d**: right lower AL at T1, **e**: left lower AL at T1, **f**: total AL at T1. Each point represents the mean (x-value) and difference (y-value) between measured and calculated AL of one arch

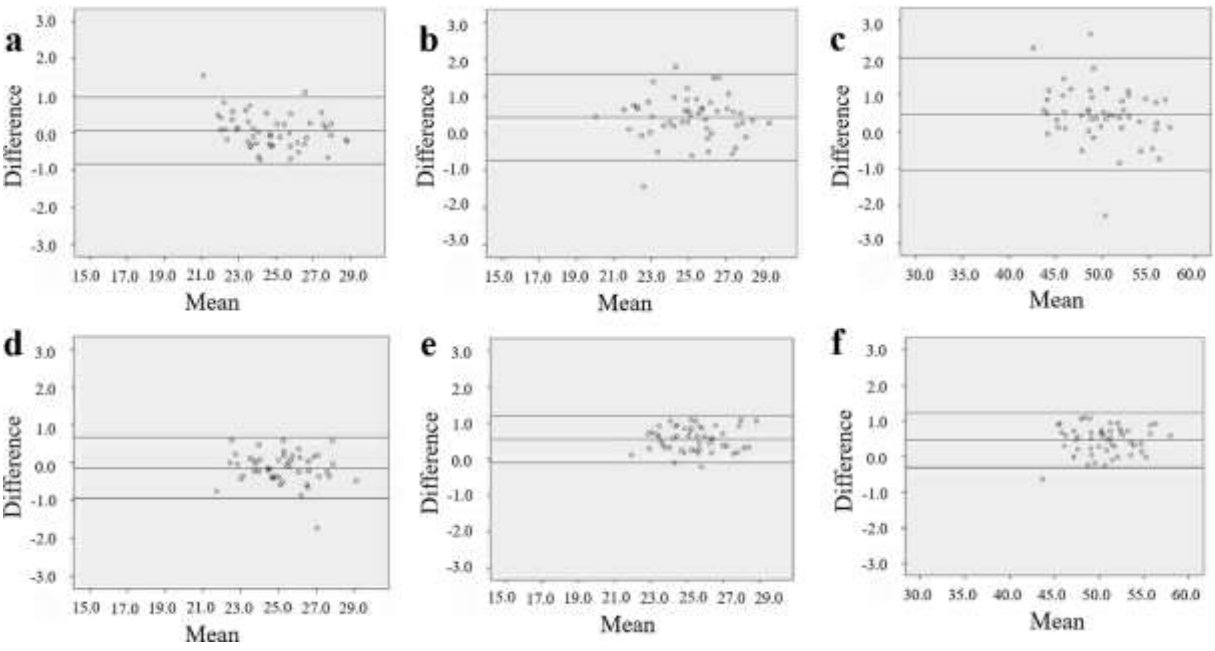

Supplementary Figure 3 Bland-Altman plots showing the difference between calculated and measured arch length (AL) in the lower jaw. **a:** right AL at T0, **b:** left AL at T0, **c:** total AL at T0, **d:** right lower AL at T1, **e:** left lower AL at T1, **f:** total AL at T1. Mean refers to the mean value of measured and calculated arch length of the same cast. Each point represents the mean (x-value) and difference (y-value) between measured and calculated AL of one arch

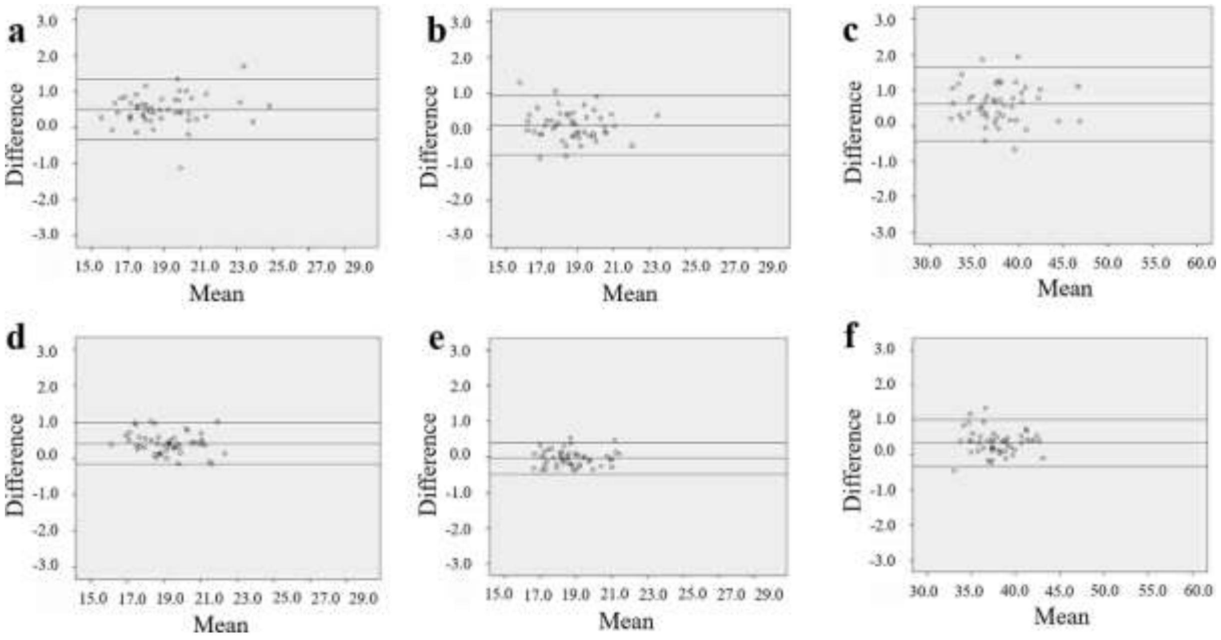

Supplementary Table 1 Impact of errors in placement of the occlusal place (OcP). Variations of the occlusal plane (OcP) of up to 6° result in changes of the arch length (AL), that do not exceed 0.1 mm and hence the potential error induced by false OcP-identification is not relevant for the clinical validation of the mathematical-geometrical model presented

**Parameters:** ML-NL = 25°, Δ∢1/NL = 3°, Δ∢1/ML = 3°, c(lj) = 20.3mm, c(uj) = 22.2mm, ∢1/ML = 92°, ∢1/NL = 70°, aaw(uj, lj) = 38.2mm, L(lj) = 16.4mm, L(uj) = 18.4mm, AL_{T0}(lj) = 54.86mm, AL_{T0}(uj) = 58.00mm					
**OcP-ML [°]; OcP-NL [°]**	**AL_{T1}(lj) [mm]**	**ΔAL(lj) [mm]**	**AL_{T1}(uj) [mm]**	**ΔAL(uj) [mm]**	**Δv(total) [mm]**
17; 8	56.41	1.55	59.63	1.63	0.94
16.5; 8.5	56.42	1.56	59.62	1.62	0.94
16; 9	56.42	1.56	59.62	1.61	0.94
15.5; 9.5	56.43	1.57	59.61	1.60	0.94
15; 10	56.43	1.57	59.60	1.60	0.94
14.5; 10.5	56.43	1.58	59.59	1.60	0.94
14; 11	56.44	1.58	59.58	1.58	0.94
13.5; 11.5	56.44	1.59	59.57	1.57	0.94
13; 12	56.45	1.59	59.56	1.56	0.94
12.5; 12.5	56.45	1.59	59.55	1.55	0.94
12; 13	56.46	1.6	59.55	1.54	0.94
11.5; 13.5	56.46	1.6	59.54	1.53	0.94
11; 14	56.46	1.61	59.53	1.52	0.94
